# Increased functional connectivity common to symptomatic amyotrophic lateral sclerosis and those at genetic risk

**DOI:** 10.1136/jnnp-2015-311945

**Published:** 2016-01-05

**Authors:** Ricarda A L Menke, Malcolm Proudfoot, Joanne Wuu, Peter M Andersen, Kevin Talbot, Michael Benatar, Martin R Turner

**Affiliations:** 1 Nuffield Department of Clinical Neurosciences, University of Oxford, Oxford, UK; 2 FMRIB Centre, John Radcliffe Hospital, University of Oxford, Oxford, UK; 3 Miller School of Medicine, University of Miami, Miami, Florida, USA; 4 Department of Pharmacology and Clinical Neuroscience, Umeå University, Umeå, Sweden

## Abstract

**Objective:**

To discern presymptomatic changes in brain structure or function using advanced MRI in carriers of mutations predisposing to amyotrophic lateral sclerosis (ALS).

**Methods:**

T1-weighted, diffusion weighted and resting state functional MRI data were acquired at 3 T for 12 asymptomatic mutation carriers (psALS), 12 age-matched controls and affected patients with ALS. Cortical thickness analysis, voxel-based morphometry, volumetric and shape analyses of subcortical structures, tract-based spatial statistics of metrics derived from the diffusion tensor, and resting state functional connectivity (FC) analyses were performed.

**Results:**

Grey matter cortical thickness and shape analysis revealed significant atrophy in patients with ALS (but not psALS) compared with controls in the right primary motor cortex and right caudate. Comparison of diffusion tensor metrics showed widespread fractional anisotropy and radial diffusivity differences in patients with ALS compared to controls and the psALS group, encompassing parts of the corpus callosum, corticospinal tracts and superior longitudinal fasciculus. While FC in the resting-state sensorimotor network was similar in psALS and controls, FC between the cerebellum and a network comprising the precuneus, cingulate & middle frontal lobe was significantly higher in psALS and affected ALS compared to controls.

**Conclusions:**

Rather than structural brain changes, increased FC may be among the earliest detectable brain abnormalities in asymptomatic carriers of ALS-causing gene mutations. With replication and significant refinement, this technique has potential in the future assessment of neuroprotective strategies.

## Introduction

Mounting evidence from the fields of Alzheimer's disease, Parkinson's disease and Huntington's disease (HD) supports the conclusion that neurodegenerative diseases are characterised by a presymptomatic phase during which pathological processes at the molecular, cellular and perhaps network levels accumulate prior to the appearance of clinically manifest disease. The same is believed to be true of amyotrophic lateral sclerosis (ALS),^1^ but evidence to this effect has been slow to accumulate and controversy persists as to the duration of such a phase. In part, this is a consequence of the difficulties inherent to studying ALS prior to the appearance of clinically apparent disease, most notably the challenge of identifying people at risk for ALS and the paucity of biomarkers that are sufficiently sensitive to permit quantification of disease in the presymptomatic state.

Over recent years, an approach has been developed and refined for studying presymptomatic ALS through the longitudinal and systematic evaluation of asymptomatic carriers of pathogenic mutations linked to the development of ALS.^1^ Importantly, the utility of biomarkers identified to be sensitive to pathology during the presymptomatic state extends beyond simply providing profound insight into the biology of this complex and devastating disorder. In addition, they present an opportunity to use the quantification of presymptomatic disease progression as a pharmacodynamic biomarker of treatment effect in disease prevention trials.^2^ Similar approaches have been successful in HD, for example, where striatal atrophy is apparent and progressive during the premanifest stage of disease, and has now been used as a surrogate end point in a small phase II disease prevention trial.^3^

With the growing evidence that advanced neuroimaging techniques are sensitive to the brain and spinal cord pathology of ALS,^4^ a range of structural analyses on T1-weighted MRI and Diffusion Tensor Imaging (DTI) data, as well as rs-fMRI analysis, was applied to a group of asymptomatic carriers of ALS gene mutations. We compared them, in parallel with a group of affected patients, to healthy controls in order to explore the potential of advanced MRI as a source of presymptomatic biomarkers in ALS.

## Methods

### Participants

Asymptomatic gene carriers, with the exception of one local participant from the Oxford Study for Biomarkers in MND (‘BioMOx’), were all participants in the Pre-Symptomatic Familial ALS (*Pre-fALS*) study (MB, JW) at the University of Miami, who travelled to Oxford University for this add-on MRI study. All were genotyped (PMA) as carriers of dominant *SOD1* mutations (A4V (n=8), I113T (n=1), N139K (n=1)); or *C9ORF72* repeat expansions (n=2). Their presymptomatic state was evidenced by the absence of symptoms, a normal neuromuscular examination, a normal electromyographic study that included evaluation of arm and leg muscles bilaterally, thoracic paraspinal muscles and bulbar musculature, and no evidence of cognitive or behavioural dysfunction on neuropsychological testing (MB and MRT). Established cases of ALS were selected from the BioMOx study, diagnosed with ALS in a tertiary referral clinic according to standard criteria (MRT and KT). All affected patients with ALS in the present study were apparently sporadic (ie, reported no family history of ALS or frontotemporal dementia). Healthy controls were a mixture of spouses and friends of affected patients, with no significant medical conditions. In order to achieve the best possible age matching, the ALS group included a higher proportion of male participants than the other two groups. Demographic and clinical characteristics of all participants who were included in the study are summarised in table 1.

**Table 1:** Demographic and clinical information for all study participants

	CON (n=12)	psALS (n=12)	ALS (n=12)
Age (years)	47.8±12.6 (20–65)	47.8±12.4 (20–65)	47.8±8.5 (31–64)
Gender	10 F: 2 M	10 F: 2 M	6 F: 6 M
Gene mutation	NA	10 SOD1 2 C9ORF72	NA
Site of symptom onset	NA	NA	2 Bulbar 2 RUL, 1 LUL, 5 RLL, 2 LLL
ALSFRS-R	NA	NA	33.8±4.5 (21–38)
Disease duration from symptom onset (months)	NA	NA	47.0±35.1 (13–123)
Progression rate (48—ALSFRS-R / duration in months)	NA	NA	0.47±0.29 (0.11–0.94)

Values are given as mean±SD (range).

ALSFRS-R, revised Amyotrophic Lateral Sclerosis Functional Rating Scale; CON, control; LLL, left lower limb; LUL, left lower limb; NA, not applicable; RLL, right lower limb; RUL, right upper limb.

Ethics committee approval for the study was granted by the South Central Oxford Ethics Committee (08/H0605/85), with written informed consent obtained from all participants. *Pre-fALS* participants were originally recruited under the authority of the Institutional Review Board of the University of Miami, USA (20101021).

### MRI data acquisition

All study participants were scanned at the Oxford Centre for Clinical Magnetic Resonance Research using a 3 T Siemens Trio scanner (Siemens AG, Erlangen, Germany) with a 12-channel head coil. High-resolution three-dimensional whole-brain T1-weighted MRI scans were acquired using a magnetisation-prepared rapid gradient echo (MPRAGE) sequence (TR/TE=2040 ms/4.7 ms, flip angle=8°, 1 mm isotropic resolution, 6 min acquisition time). Whole-brain diffusion-weighted images were acquired using an echoplanar sequence (60 isotropic directions; b value=1000 s/mm^2^; echo time/repetition time=94 ms/10 000 ms; 2×2×2 mm^3^ voxel size; 65 slices). In addition, four images without diffusion weighting were acquired. Whole-brain functional imaging at rest was performed using a gradient echo Echo Planar Imaging (EPI) sequence (TR/TE=3000/28 ms, flip angle=89°, 3 mm isotropic resolution, 6 min acquisition time). For consistency, participants were instructed to close their eyes throughout, but to remain awake. Furthermore, a field map was acquired using a gradient echo imaging sequence (2×2×2 mm^3^ voxel size; 65 slices; echo time 1/echo time 2/repetition time=5.19 ms/7.65 ms/655 ms) to account for distortions present in the DTI and functional MRI data caused by field inhomogeneities.

### MRI data analysis

#### Volumetric analysis of cortical grey matter (VBM)

T1-weighted MPRAGE data were analysed with FSL-VBM, a voxel-based morphometry style analysis. A standard optimised FSL-VBM protocol was run for the structural images of all participants. First, structural images were brain-extracted.^5^ Next, tissue-type segmentation was carried out using FAST4.^6^ The resulting grey matter partial volume images were then aligned to MNI152 standard space using the affine registration tool FLIRT,^7^ followed by non-linear registration using FMRIB Non-linear Integration and Registration Tool (FNIRT).^8^ The resulting images were averaged to create a symmetric, study-specific template, to which the native grey matter images were then non-linearly re-registered. We then multiplied the registered partial volume images of all participants by the Jacobian of the warp field (‘modulation) to correct ‘for local expansion or contraction. The modulated segmented images were then smoothed with an isotropic Gaussian kernel with a sigma of 3 mm.

The Juelich Histological Atlas was used to produce masks of the frontal lobes and of the left and right primary motor cortices (PMC). To assess group differences (‘two groups, unpaired’ test as implemented in FMRIB Software Library (FSL)), a voxel-wise generalised linear model (GLM) was applied using permutation-based non-parametric testing. Results with p<0.05 were considered significant, after correction for multiple comparisons (familywise error, FWE) within each region of interest (whole grey matter as well as masks derived from the Juelich Histological Atlas), using the threshold-free cluster enhancement (TFCE) approach.^9^ Differences with the psALS group were assessed only for those contrasts that showed significant differences between patients and controls.

#### FreeSurfer cortical thickness (CT) analysis

Individual cortical parcellations were obtained using FreeSurfer (V.5.2). Individual participants’ T1 volumes were linearly aligned to the MNI 305 average brain template, bias corrected, skull stripped and segmented into tissue types. The segmented white matter volume was used to derive a surface representing the grey-white matter boundary, which was automatically corrected for topology defects and carefully inspected in each participant for accurate tissue classification. The grey-white surface was inflated to form a sphere and warped to match curvature features across participants.^10^ ^11^ After alignment to the spherical-space standard curvature template, the cortex was partitioned on the basis of a gyral and sulcal structure using an automated segmentation procedure.^12^ Group differences of average CT of the left and right Brodman Area (BA) 4 (anterior and posterior) and BA6 were assessed by one-way analysis of variance (ANOVA) (as implemented in IBM SPSS Statistics, V.20) for Mac. Post hoc statistical comparisons between controls, psALS participants and patients with ALS were carried out using ‘Least Significant Difference’ (LSD) post hoc tests. The two-sided significance level was set at 0.05. Furthermore, vertex-wise CT was compared pairwise between the three groups using *mri_glmfit,* part of the *FreeSurfer* toolkit. Inference was performed using permutation testing, allowing correction for multiple comparisons False Discovery Rate (FDR) across surface vertices. The significance threshold was set at p_FDR_<0.05. Differences with the psALS group were assessed only for those contrasts that showed significant differences between patients and controls.

#### Volumetric and shape analysis of subcortical grey matter structures (FIRST)

We segmented the putamen, caudate, pallidum, thalamus, amygdala and hippocampus from each participant's MPRAGE image using FMRIB’s Integrated Registration and Segmentation Tool (FIRST).^13^ The results of the subcortical segmentation were carefully examined to ensure accuracy of the results.

For each participant, whole brain tissue volume, normalised for participant head size, was estimated with SIENAX,^14^ part of FSL. SIENAX starts by extracting brain and skull images from the single whole-head input data. The brain image is then affine-registered to the MNI152 space (using the skull image to determine the registration scaling); this is primarily in order to obtain a volumetric scaling factor to be used for normalisation for head size as described below.

Before conducting statistical analyses, the volumes of each subcortical region of interest were normalised for head size via multiplication by the volumetric scaling factor derived from SIENAX. All statistical analyses were carried out using IBM SPSS Statistics (V.20) for Mac. Volumes of all segmented regions were assessed for group differences by one-way ANOVA. *Post hoc* statistical comparisons between psALS participants, affected patients with ALS and healthy controls were carried out using ‘Least Significant Difference’ (LSD) post hoc tests. The two-sided significance level was set at 0.05.

Vertex analysis was performed using FIRST in a mode of operation that aims to assess group differences on a per-vertex basis (the meshes were reconstructed in native space). To assess group differences (‘two groups, unpaired’ test as implemented in FSL) between controls, psALS participants and age-matched patients WITH ALS, voxel-wise GLM was applied using permutation-based non-parametric testing. Results with p<0.05 were considered significant, after cluster-based multiple-comparison-correction. Differences with the psALS group were assessed only for those contrasts that showed significant differences between patients and controls.

#### DTI preprocessing

Each participant's DTI scans were corrected for head motion and eddy currents^7^ and then brain-extracted to remove any non-brain voxels. To correct for B_0_ inhomogeneities and unwarp scans, field map correction was performed with FUGUE. Fractional anisotropy (FA), mean diffusivity (MD), axial diffusivity (eigenvector L1) and L2 and L3 maps were created using DTIFIT by applying a diffusion tensor model to each voxel.^15^ Radial diffusivity (RD) maps were created by averaging the L2 and L3 maps (RD=(L2+L3)/2).

#### Tract-based spatial statistics (TBSS) preprocessing and statistical analysis

All individual FA images of all participants were non-linearly registered to a standard FA template (http://fsl.fmrib.ox.ac.uk/fsl/fslwiki/FMRIB58_FA), and then averaged to create a study-specific template to which each participant’s FA map was then non-linearly registered. Next, the mean FA image was created and thinned to create a mean FA skeleton,^16^ which represents the centre of all tracts common to the group. Each participant’s aligned FA data were then projected onto this skeleton**.** The same operations that were used to register the individual FA images to the study-specific template and project FA values onto the mean FA skeleton were subsequently applied to the individual MD, L1, L2 and L3 images.

The Juelich Histological Atlas was used to produce masks of major tracts that have previously been shown to be involved chiefly in ALS, namely the left and right corticospinal tracts (CST), superior longitudinal fascicle (SLF), body of corpus callosum (CC),^17^ as well as the uncinate fasciculus and the cingulum, which were derived from the JHU white-matter tractography atlas, in the Montreal Neurological Institute (MNI) standard space. Left and right CST and CC masks were thresholded at ‘45%’ and the left and right SLF masks were thresholded at ‘10%’. Masks of the left and right uncinate fasciculi and the cingulum bundles were thresholded at ‘20%’. The final region-of-interest (ROI) resulted from the intersection of the atlas-based masks and the mean FA skeleton mask.

To assess group differences (‘two groups, unpaired’ test as implemented in FSL) between controls, psALS participants and age-matched patients, voxel-wise GLM was applied using permutation-based non-parametric testing. Results with p<0.05 were considered significant, after correction for multiple comparisons (familywise error, FWE) within each region of interest (whole brain as well as masks derived from the Juelich Histological Atlas), using the TFCE approach.^9^ Differences with the psALS group were assessed only for those contrasts that showed significant differences between patients and controls.

#### Resting state functional MRI

Resting-state analysis was performed using probabilistic independent component analysis (ICA) as implemented in the FSL tool Multivariate Exploratory Linear Optimized Decomposition into Independent Components (MELODIC).^18^ The ICA approach was favoured over seed-based correlation analysis because it enables automated isolation of resting-state brain networks (RSNs) where individual areas in a given network are tightly functionally connected and avoids seed selection bias.^19^ Individual preprocessing consisted of motion correction, brain extraction, unwarping using fieldmap data, spatial smoothing using the Gaussian kernel of FWHM of 6 mm, and high-pass temporal filtering of 150 s. To correct for motion, physiological noise and other artefacts, we employed a previously described ICA-based de-noising approach.^20^ In summary, after performing participant-level ICA with automated dimensionality estimation, the FIX tool (http:// http://fsl.fmrib.ox.ac.uk/fsl/fslwiki/FIX) was used to automatically classify the obtained components into signal or noise, and the noise contribution was regressed out from the data. Subsequently, data were linearly registered to the corresponding structural image using FLIRT, optimised using Boundary-Based Registration,^21^ and subsequently registered (via the structural image) to the MNI space using non-linear registration.

A group template of independent components (ie, RSNs) was then generated from all 36 participants via ‘multi-session temporal concatenation’ as implemented in MELODIC (the number of output components was limited to ‘20’). The dual regression approach^22^ was then used to identify individual temporal dynamics and the associated spatial maps for all RSNs of interest. In brief, spatial regression was performed using the spatial maps obtained from the resting state template in a GLM against each participant's individual fMRI data, which resulted in participant-specific time courses for each of the components. Then, these time courses were used in a further GLM to generate participant-specific spatial maps for each RSN.

Group comparisons between affected patients with ALS and controls were then performed using permutation-based non-parametric inference within the framework of the GLM (‘randomise’). All analyses with p<0.05 were considered significant after FWE rate (TFCE^9^) correction. Only RSNs for which significant differences between patients and control participants were observed were subsequently assessed for differences between psALS participants and the other two groups.

## Results

### Study participants

Thirteen psALS participants underwent MRI, but one participant had to be excluded from the TBSS analyses due to artefacts in the diffusion-weighted data. MRI data were therefore available for 12 psALS participants. Twelve patients with ALS and 12 control participants were chosen from the larger BioMOx cohort to ensure the best possible age and gender matching with the psALS group. One female control participant who was included in the VBM, FreeSurfer and resting state functional MRI (rs-fMRI) analyses had to be replaced by a male participant (in order to preserve optimal age matching) for the TBSS analyses because of artefacts in her diffusion-weighted data. Furthermore, automated segmentation of subcortical structures failed for two female psALS participants, who were therefore excluded from the FIRST analyses.

### VBM and CT analyses

VBM analyses (n=12 per group) did not reveal any significant group differences (ALS vs control) for any of the ROIs. Furthermore, vertex-wise CT comparisons using FreeSurfer did not reveal any significant group differences after correction for multiple comparisons across vertices.

**Figure 1:**
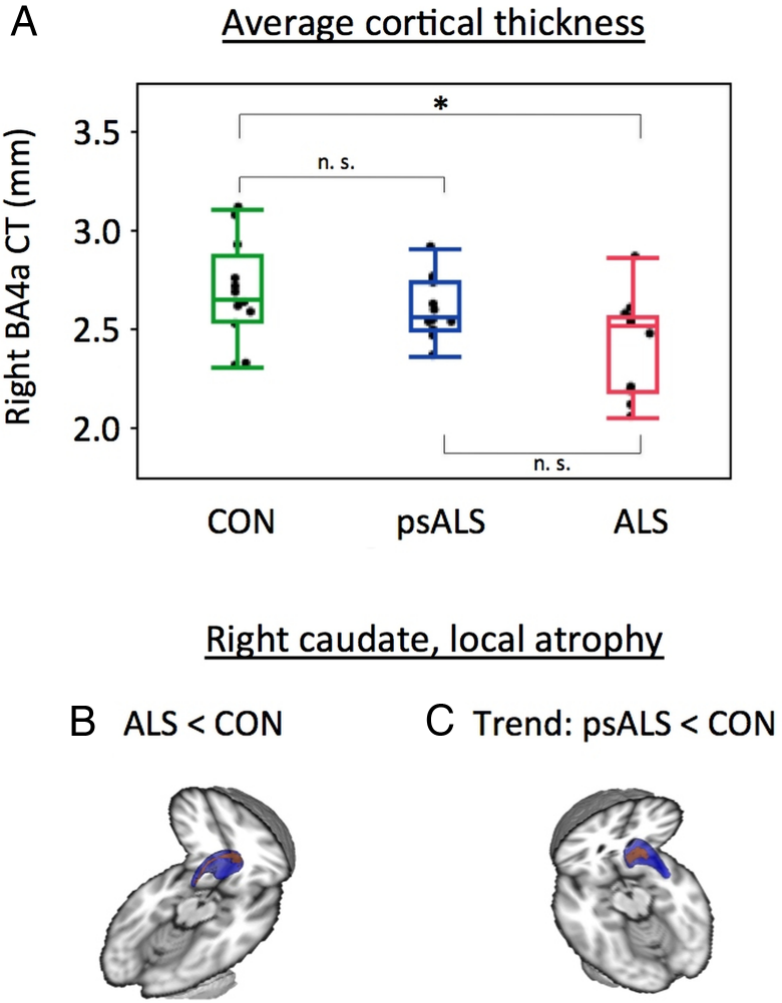
Grey matter pathology. (A) Thickness of the right anterior primary motor cortex (BA4a) was reduced in patients with ALS compared to controls (p=0.008); the psALS group did not significantly differ from the other two groups. (B) Focal atrophy of the right caudate nucleus in patients with ALS compared to control participants (structure shown in blue, results for p_FWE_<0.05 shown in red). (C) Atrophy of the right caudate nucleus in psALS compared to controls (red, p_FWE_=0.074). BA, Brodman Area; _FWE,_ familywise error.

The results of the average CT analyses revealed a significantly lower thickness in patients with ALS compared to controls for the right anterior PMC (BA 4a) (p=0.008) (figure 1A). Although there were no significant differences between the psALS and any of the other two groups, the psALS group's average CT values for the right anterior PMC were intermediate between measures for patients and controls.

### Volumetric and vertex-wise analysis of subcortical structures

Vertex-wise analysis revealed significant shape differences for the right caudate between patients with ALS and controls (n=10 per group, indicating local atrophy in patients, figure 1B) (p_FWE_<0.05), with a trend for the comparison between controls and psALS participants (p_FWE_=0.074) for the same structure (figure 1C).

### TBSS analysis

**Figure 2:**
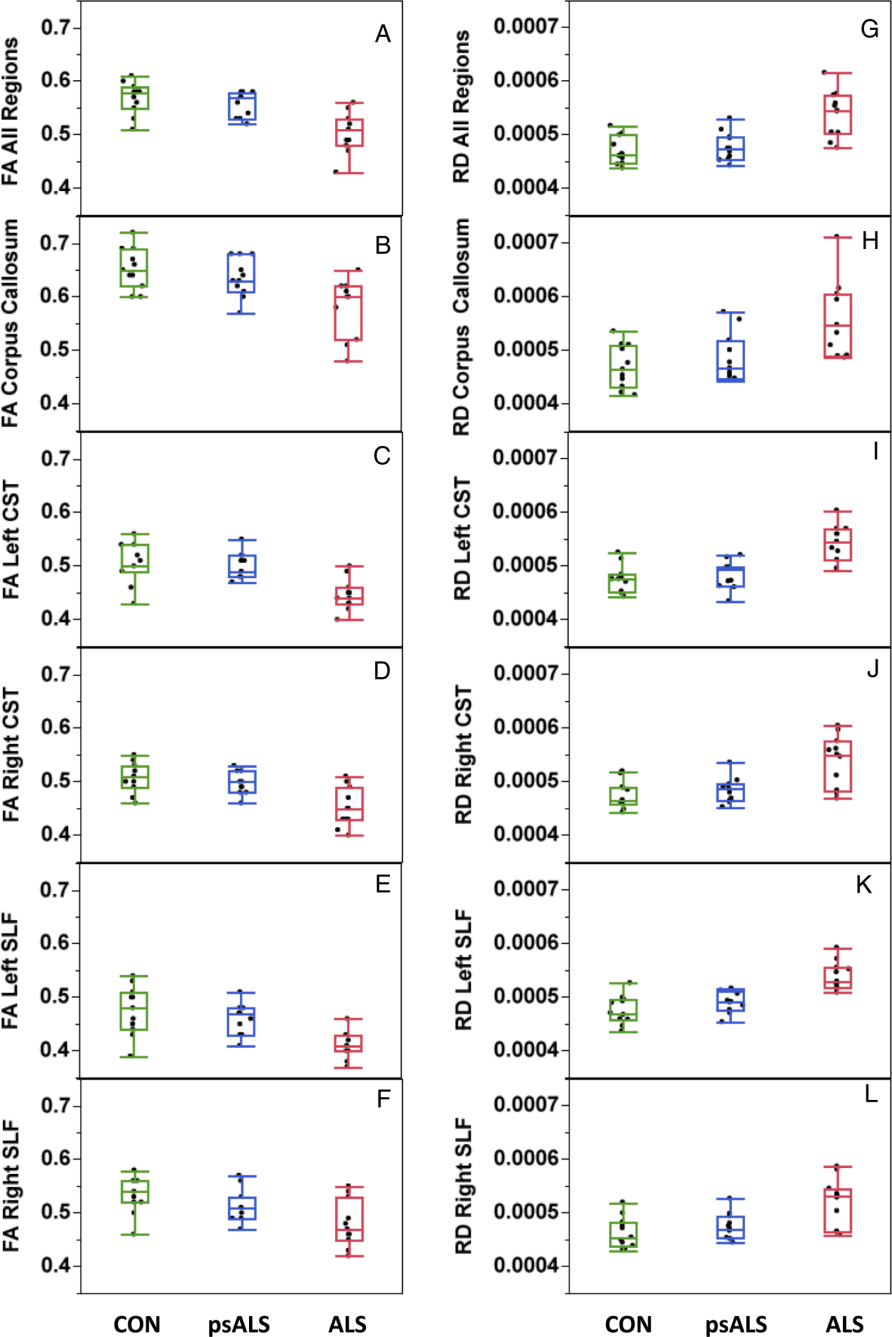
White Matter Pathology. Regions of significant difference in (A) FA and (B) RD between patients with ALS and controls (ie, significance masks) in a TBSS analysis encompassing the major tracts shown to be involved in ALS—the corpus callosum, the CST bilaterally, and the SLF bilaterally. (B, C, D, E, F) illustrate, in each tract, the differences in FA (within the significance masks comparing patients with ALS to controls) across the three participant groups. (H, I, J, K, L) illustrate, in each tract, the differences in RD (within the significance masks comparing patients with ALS to controls) across the three participant groups. ALS, amyotrophic lateral sclerosis; CST, corticospinal tracts; FA, fractional anisotropy; SLF, superior longitudinal fasciculus; RD, radial diffusivity; TBSS, tract-based spatial statistics.

Using the entire white matter skeleton as an ROI, widespread differences in FA and RD were observed, encompassing parts of the CC, CSTs and SLFs in patients with ALS compared to controls and the psALS group, but not between controls and psALS (n=11 per group) (figure 2A, B). Further examination of each of the CC, CSTs and SLFs tracks revealed that, despite the fact that psALS FA and RD values in these regions were always intermediary between FA and RD values of affected ALS and controls (figure 2C–L), there were no *significant* differences between the psALS and control groups in regions that were found to show significant differences between patients and controls.

Analyses confined to the uncinate fasciculi and the cingulum bundles did not reveal any significant differences between patients and controls.

### Resting state fMRI

**Figure 3:**
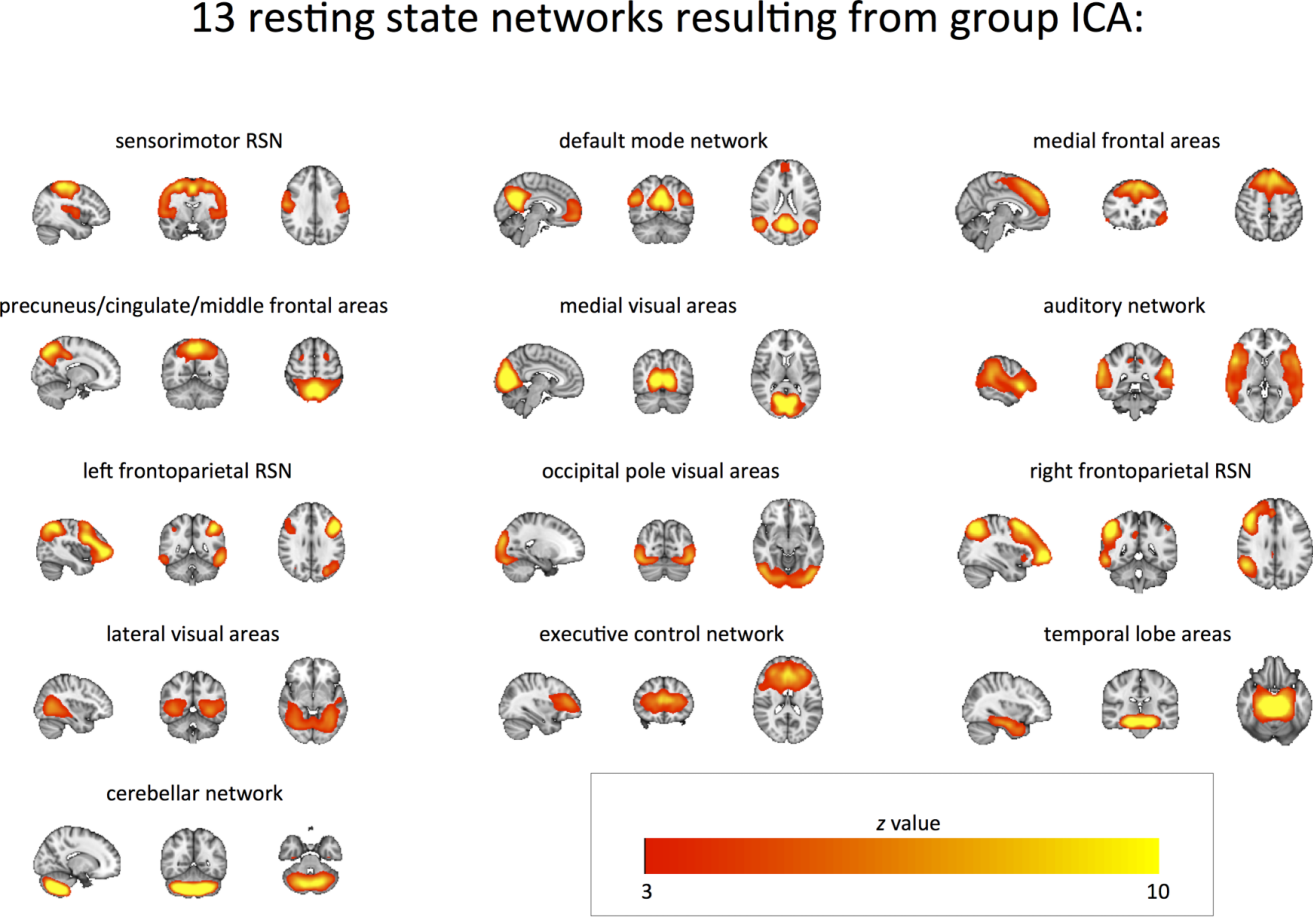
Resting State Functional MRI Networks. Resting state networks (red-yellow) obtained via group ICA of resting state MRI data of all participants. ICA, independent component analysis.

Out of the 20 RSNs that resulted from the ‘multi-session temporal concatenation’ described above, seven were discarded as ‘noise components’. The 13 remaining RSNs of interest corresponded to the sensorimotor; default mode; medial frontal; precuneus-cingulate-middle frontal (P-C-MF); medial visual; occipital pole; lateral visual; auditory; the left and right frontoparietal; executive control; temporal lobe; and cerebellar RSNs (figure 3).

**Figure 4:**
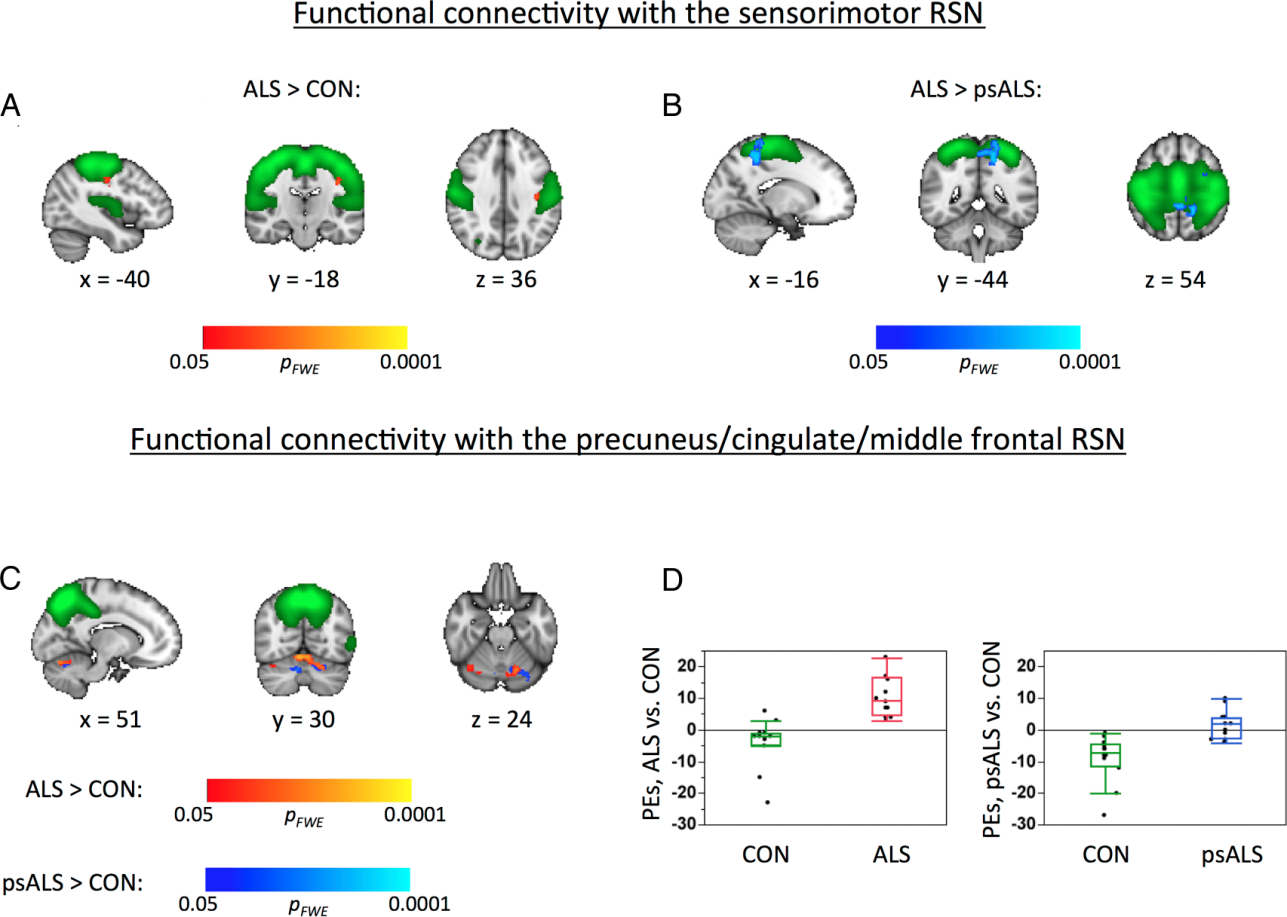
Functional connectivity. Within the sensorimotor RSN, there was increased FC in (A) the ALS patient group compared to controls, and (B) in the ALS patient group compared to the psALS group. (C) FC within the P-C-MF RSN was increased in both the ALS patient and psALS groups compared to controls. Results (shown in red-yellow and blue colours) are overlaid onto the respective RSNs (thresholded at z>3, green) and the T1-weighted MNI template (greyscale). x/y/z=MNI coordinates. Using significance masks for areas of increased FC in the P-C-MF RSN between patients with ALS and controls, (D) illustrates differences in FC within this RSN for patients with ALS compared to controls (left panel) and psALS participants compared to controls (right panel). ALS, amyotrophic lateral sclerosis; FC, functional connectivity; P-C-MF, precuneus-cingulate-middle frontal; RSN, resting state network.

Significant group differences (n=12 per group) were detected between affected patients with ALS and controls for two RSNs, namely sensorimotor and the P-C-MF RSN. There was *increased* functional connectivity (FC) between a small area of the left primary motor cortex and the rest of the sensorimotor RSN (figure 4A), and *increased* FC between parts of the cerebellum and the P-C-MF RSN (figure 4C).

Comparison of psALS participants with affected patients with ALS and controls for these two RSNs revealed significantly *increased* FC in affected patients with ALS compared to the psALS group for the sensorimotor RSN (figure 4B). For the P-C-MF RSN, significantly *higher* FC with the cerebellum was observed in the psALS group compared with controls, regionally similar to that seen between affected patients with ALS and controls (figure 4C, blue). No significant FC differences between the psALS group and affected patients with ALS were found for this network.

**Figure 5:**
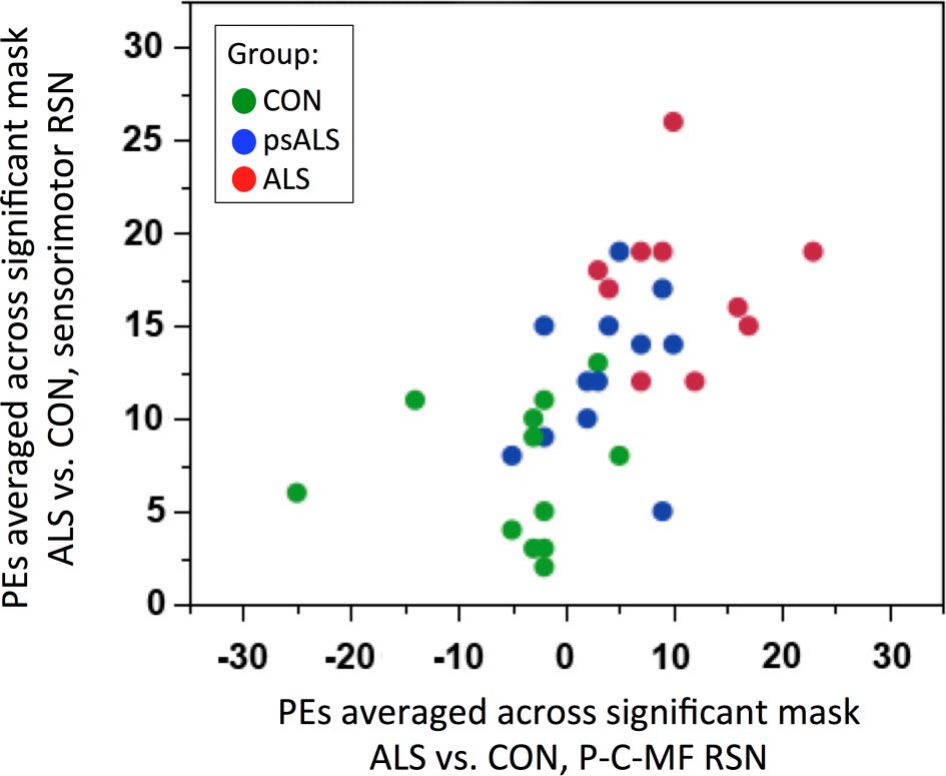
Functional connectivity discrimination. Distribution of PE extracted from sensorimotor (y axis) and the P-C-MF (x axis) RSNs, areas that show significantly increased FC between patients with ALS and healthy controls, across the three groups. ALS, amyotrophic lateral sclerosis; FC, functional connectivity; P-C-MF, precuneus-cingulate-middle frontal; PEs, parameter estimates; RSN, resting state network.

Utilising parameter estimates extracted from the sensorimotor and the P-C-MF networks (ie, areas that show significant FC differences between control participants and patients with ALS), a clear separation between patients with ALS and controls was apparent. Importantly, the psALS participants appeared intermediate between the patients with ALS and controls, and overlapped with both groups (figure 5).

## Discussion

We have assessed the potential of different MRI modalities and analysis strategies to detect presymptomatic brain changes in those at high risk of developing ALS. The important observations in a genetically and demographically heterogeneous asymptomatic group compared to age-matched controls were:

- There were no significant differences in structural grey or white matter measures.
- Functional connectivity between the P-C-MF network and the cerebellum was increased, to a similar degree as that observed in affected patients.

It must be acknowledged as a potential source of bias that, owing to the limited availability of participants, the groups in this study were matched for age, but not for gender.

### When does ALS begin?

Like all human organs, the brain and associated nervous system have high levels of functional reserve, so pathology is likely to be established long before symptoms become apparent. To the patient with ALS, there is often a strikingly abrupt onset to their weakness. Both lower motor neuron studies involving motor unit number estimation,^23^ and upper motor neuron studies involving measurement of cortical excitability using transcranial magnetic stimulation in presymptomatic *SOD1* mutation carriers,^24^ suggest that loss of motor units and increased cortical excitability, respectively, are detectable only a few months prior to the onset of symptoms. However, it cannot be inferred that there are no pathological changes prior to these *measurable* events.

Understanding the earliest functional consequences of molecular changes will be vital to assessing future neuroprotective therapies. As well as the consideration of primary prevention strategies in individuals at risk for genetic forms of ALS, the development of pharmacodynamic biomarkers of subclinical motor system dysfunction has great relevance to ALS cases that are apparently sporadic, for whom the current standard outcome measures in therapeutic trials are survival and functional status.

### Structural MRI abnormalities

Only modest grey matter atrophy limited to the right anterior primary motor cortex and the right caudate was seen in the affected patient group compared to controls. This is in line with previous reports of limited grey matter involvement in ALS from cross-sectional studies in larger cohorts.^25^ ^17^ The more obvious DTI-based white matter tract pathology, namely regions of decreased FA and increased RD in the CSTs, CC and SLFs of the affected patient group, has all been consistently reported in the past.^4^

Against this background of limited grey matter structural changes in the established ALS brain, analysis of grey matter volumes, density or shape in psALS did not reveal any significant differences compared to controls. While this is in keeping with current models of Alzheimer's disease, where detectable grey matter volumetric MRI changes are thought to occur just prior to the development of symptoms,^26^ significant cortical changes were detected in a study of asymptomatic carriers of hexanucleotide expansions in *C9orf72* at high risk of MND and FTD.^27^ White matter, but not grey matter changes were found in another study of presymptomatic FTD participants.^28^ In relation to presymptomatic ALS, while one previous study reported reduced FA in the posterior limb of the internal capsules in a small sample of asymptomatic *SOD1* mutation carriers,^29^ this finding was not replicated in a second study,^30^ nor in this study. It is, however, noteworthy that, despite not reaching significance in comparison to control data, the values observed in the psALS group were qualitatively ‘intermediate’ for all regions in which we found significant grey or white matter differences between patients with ALS and controls.

### Functional MRI abnormalities

Within the sensorimotor network, FC was significantly higher in the affected patients with ALS compared to control participants, while the psALS group scores were comparable to controls. In contrast, cerebellar FC with the P-C-MF RSN was significantly increased in psALS participants in comparison to controls, revealing abnormalities that were similar to those found in the affected patients, in terms of scores and localisation. Subclinical involvement of the cerebellum has been reported in *SOD1* ALS.^31^ The lack of overt ataxia or nystagmus in ALS more widely has possibly led to the neglect of this brain region in imaging studies, despite its critical involvement within the greater motor system. Relevant MRI findings in ALS include increased motor task-related cerebellar fMRI activation,^32^ increased cerebellar FC with sensorimotor areas in ALS,^33^ hyperconnected subcortical motor networks spanning the basal ganglia and cerebellum,^34^ and abnormally decreased FA within the culmen that correlated with scores of disease severity and executive functioning.^35^ A distinct cerebellar pathology has been highlighted in patients with ALS with intronic expansions in *C9ORF72.*^36^ Although our psALS group included two *C9ORF72* gene mutation carriers, their respective FC scores were among those closest to the scores found in controls, and so seem unlikely to solely account for our observations of cerebellar involvement.

The observations of *increased* FC in established ALS might reflect either a compensatory plasticity,^37^ or be driven by a loss of local inhibitory neuronal circuits,^22^ ^38^ which then overlaps with the established concept of cortical excitability. Our results do not allow the firm conclusion that the functional brain changes occur *earlier* than structural white or grey matter abnormalities in ALS pathogenesis, and might simply reflect the differing sensitivity of current MRI sequences, especially in the context of the clinical heterogeneity (eg, variable expected latency to onset of manifest disease) inherent to cross-sectional studies in asymptomatic individuals such as reported here. It is also noted that the group age-matching process resulted in having to select patients with ALS with a relatively low mean rate of disease progression, which may have further reduced the sensitivity to group differences.

### Genotype heterogeneity and penetrance

Although the expanding clinical syndrome of ALS still has a set of core features recognisable to the neurologist, the discovery of diverse predisposing genes in patients with similar clinical manifestations means it is axiomatic that there are multiple upstream pathways feeding into the final common pathway of progressive motor neuron loss. The psALS group consisted of mainly, though not completely, *SOD1* mutation carriers who also differed in their pathological mutations, as well as age. While the eventual clinical presentation may be shared with sporadic ALS, the early neuropathological processes may be different both within gene (ie, between different *SOD1* mutations) as well as between genes (*SOD1* vs *C9ORF72*). The former has been hinted at by a previous DTI study in affected patients with ALS homozygous for the recessive and more slowly progressive D90A *SOD1* mutation, which revealed a relative sparing of CST white matter involvement compared with patients with sporadic ALS matched for disability and clinical upper motor neuron (UMN) involvement.^39^

The penetrance of *SOD1* mutations and *C9orf72* expansions is not complete, so our psALS group is not necessarily ‘pre-symptomatic’. However, the mean age of symptom onset for most carriers of *SOD1* mutations (which makes up most of the group) is 47,^40^ which is the mean age of the psALS group. It is also entirely possible that there are MRI or other abnormalities detectable in individuals who will never develop fulminant symptomatic disease, though this remains speculative at present.

Another potential concern relates to the difference in histopathology between apparently sporadic cases of ALS (characterised by cytoplasmic inclusions of ubiquitinated TDP-43) and *SOD1*-mediated ALS, which appears to lack TDP-43 inclusions.^41^ While this might then weaken inferences about similar RSN involvement in our affected and psALS groups versus controls, in fact there are similar aggregates of misfolded *SOD1* species across a range of autopsied patients with ALS, suggesting that at least part of the neurodegenerative pathways are shared between different subtypes of ALS.^42^ ^43^ In a larger study of asymptomatic carriers of FTD-associated gene mutations, more extensive white matter changes were noted in a separate analysis of *MAPT* and *GRN* mutation groups versus controls, although clear functional changes were observed across the pooled group.^28^

In conclusion, despite clear caveats, our results indicate that FC measures derived from rs-fMRI may reflect presymptomatic ALS cerebral pathology. Biomarkers in this setting will be an essential part of the rapid assessment of future putative neuroprotective agents.
